# The Role of Osteopontin in the Pathogenesis and Complications of Type 1 Diabetes Mellitus in Children

**DOI:** 10.4274/jcrpe.3082

**Published:** 2016-12-01

**Authors:** Mohamed A. Talat, Laila Metwaly Sherief, Hosam Fathy El-Saadany, Anwar Ahmed Rass, Rabab M. Saleh, Maha Mahmoud Hamed Sakr

**Affiliations:** 1 Zagazig University Faculty of Medicine, Department of Pediatrics, Zagazig, Egypt; 2 Zagazig University Faculty of Medicine, Department of Clinical Pathology, Zagazig, Egypt

**Keywords:** type 1 diabetes mellitus, osteopontin, cytokines, Microalbuminuria, retinopathy

## Abstract

**Objective:**

Type 1 diabetes mellitus (T1DM) is the most common chronic metabolic disorder of childhood and adolescence. Osteopontin plays a significant role in the development and progression of several autoimmune diseases. Moreover, osteopontin promotes adipose tissue inflammation, dysfunction, and insulin resistance. To investigate the levels of serum osteopontin in pediatric patients with T1DM and to explore if these levels have a role in the prediction of diabetes complications.

**Methods:**

This was a case–control study conducted at the Endocrinology unit of the Children’s Hospital of Zagazig University in Egypt, from October 2014 to December 2015. Sixty patients with T1DM and 60 healthy subjects were enrolled. A detailed medical history was taken from all patients/parents. A full clinical examination including ophthalmoscopy was performed on all patients. Fasting blood glucose, hemoglobin A1c (HbA1c), urine albumin/creatinine ratio, and serum osteopontin levels were also determined in all subjects.

**Results:**

Patients with T1DM had significantly higher serum osteopontin levels compared with controls (mean ± standard deviation: 13.7±3.4 μg/L vs. 8.9±2.9 μg/L, p<0.001). Also, serum osteopontin concentrations were higher in patients with microalbuminuria than in patients with normal albumin excretion rate and in the control group. Similarly, those who had retinal disease had higher osteopontin concentrations than those without (16.8±2 vs. 12.4±3 mg/L; p=0.005). Serum osteopontin levels correlated with a diagnosis of T1DM, and in diabetic patients, correlated with higher systolic and diastolic blood pressure, body mass index values and with lower high density lipoprotein values, diagnosis of retinopathy, and microalbuminuria. No correlation was found between osteopontin levels and HbA1c, insulin dose, co-medications, and diabetes duration in T1DM patients. The association between high osteopontin levels and T1DM was independent from all confounders.

**Conclusion:**

This study shows that increased osteopontin levels are independently associated with T1DM in pediatric patients and supports the hypothesis that osteopontin may have a role in the prediction of microvascular diabetes complications.

**WHAT IS ALREADY KNOWN ON THIS TOPIC?**

Type 1 diabetes mellitus (T1DM) is the most common chronic metabolic disorder of childhood and adolescence. Many patients with diabetes eventually develop physical and emotional complications, including neuropathy, nephropathy, retinopathy, and cardiovascular disease.

**WHAT THIS STUDY ADDS?**

Increased osteopontin levels are independently associated with T1DM in pediatric patients.

## INTRODUCTION

Type 1 diabetes mellitus (T1DM) is the most common chronic metabolic disorder of childhood and adolescence. It is characterized as a disorder in the metabolism of carbohydrates, lipids and amino acids as a result of decreased insulin. Many patients with diabetes eventually develop physical and psychological complications, including neuropathy, nephropathy, retinopathy, and cardiovascular disease (1).

T1DM develops as a result of an autoimmune process, leading to beta-cell destruction (2). In the early stages of insulitis, activated natural killer cells, dendritic cells, macrophages, and T-cells are attracted to the islets. This early phase is followed by production of cytokines and free radicals, which contribute to beta-cell dysfunction and death (3).

Osteopontin (OPN) is a phosphoprotein with adhesive and cell signaling functions; it can act either as an extracellular matrix component in mineralized tissue or as a soluble cytokine in inflamed tissue and serum (4). It plays a vital role in the regulation of immune cell response as it modulates T cell function by affecting the differentiation of T lymphocytes into Th1 and Th2 cells, regulating the balance between Th1 and Th2, and participating in the cell-induced immunological response (5).

OPN was demonstrated to induce adipose tissue inflammation and to increase pro-inflammatory cytokines release in the bloodstream (6). Also, itself acts as a pro-inflammatory cytokine by chemoattracting monocytes, macrophages, and lymphocytes (7). It also stimulates B lymphocytes to express multi-clone antibodies (8). Consequently, OPN promotes the destruction of pancreatic beta-cell and development of T1DM. Several authors reported that increased OPN levels were found to be a predictor of coronary calcification, nephropathy, and coronary artery disease in patients with type 2 diabetes mellitus, independent of traditional risk factors (9,10). However, there are limited data regarding the role of OPN in TIDM in children.

The aim of this study was to investigate the levels of serum OPN in pediatric patients with T1DM compared to non-diabetic participants and to explore if it has a role in the prediction of microvascular and macrovascular complications of diabetes.

## METHODS

This case-control study was conducted on 60 children with T1DM recruited from those attending the Pediatric Endocrinology Outpatient Clinic at the Children’s Hospital, Zagazig University, Egypt, from October 2014 to December 2015, fulfilling the following inclusion and exclusion criteria:

Inclusion criteria: 1) Age: less than 18 years old. 2) Gender: both males and females. 3) Insulin dependency for controlling blood sugar within normal ranges.

Exclusion criteria: 1) Conditions which may lead to insulin resistance such as obesity, acanthosis nigricans. 2) Acute inflammatory illness (including a common cold, infections) as it can affect the serum OPN level. 3) Children with end-stage renal diseases.

Sixty apparently healthy children were included as a control group.

Written informed consent was obtained from the parents of the patients involved in the study as recommended by the Institutional Ethics Committee of Zagazig University and in accordance with the Helsinki declaration after a full explanation of the purpose and nature of all procedures used.

All studied patients were subjected to the following: Detailed history taken laying stress on age, gender, symptoms of diabetes, duration of the disease, frequency of self-monitoring blood glucose (SMBG) by asking patients to estimate approximately how many times a week they usually monitored their blood glucose, recent history of infections or serious illness, daily insulin dose (IU/kg/day), response to insulin therapy, complications of diabetes or insulin therapy, and concomitant medications at the time of study enrollment [angiotensin-converting enzyme inhibitors (ACE-I)].

All subjects underwent a careful physical examination. Weight, height, systolic and diastolic blood pressure (SBP, DBP, mmHg) were measured. Body mass index (BMI) (kg/m2) was calculated and manifestation of insulin side effects were recorded in each subject.

Ophthalmoscopy was performed at the Zagazig University Hospital ophthalmology outpatient clinic by an ophthalmologist with expertise in diabetes. Ophthalmoscopy was followed by retinal fluoroangiography, when indicated. Retinal examination was used to identify and quantify diabetic retinopathy (DR) according to the International Clinical DR Disease Severity Scale (11).

All study participants underwent blood sampling for biochemistry after an overnight fasting.

Fasting blood glucose (FBG) and hemoglobin A1c (HbA1c) were measured by high-performance liquid chromatography (Tosoh 2.2; Tosoh Bioscience, South San Francisco, CA).

Blood urea nitrogen (BUN, mg/dL), creatinine (mg/dL), aspartate aminotransferase (AST, IU/L), and alanine aminotransferase (ALT, IU/L) values were estimated (Cobas Integra 400 plus, Roch Germany). Total cholesterol (mg/dL), low-density lipoprotein-cholesterol (mg/dL), high-density lipoprotein-cholesterol (mg/dL), and triglyceride (mg/dL) levels were also determined.

Microalbuminuria was estimated as the albumin/creatinine ratio in a random spot urine specimen. To this end, the first morning midstream urine samples (10 mL) were collected in sterile containers and centrifuged at 2000-3000 rpm for 20 minutes. The supernatant was removed. The urinary microalbumin and urinary creatinine were measured immediately after centrifugation. Microalbuminuria was assayed by the immunoturbidimetric method (Biosystems SA, Costa Brava, Barcelona, Spain). Creatinine was assayed by Modified Jaffes method using a fully automated Chemistry Analyzer of Cobas Integra 400 plus. Urinary microalbumin/creatinine ratio was calculated as urine microalbumin (mg)/urine creatinine (g).

Serum OPN level was measured using sandwich enzyme immunoassay technique by Enzyme-Linked ImmunoSorbent Assay kit provided by Glory Science Co., Ltd, USA.

### Statistical Analysis

Data were checked, entered, and statistically analyzed by SPSS (Statistical Package for Social Sciences version 19, Chicago, IL, USA) and were expressed as mean ± standard deviation for quantitative variables and number and percentage for categorical variables.

Student’s t-test for continuous variables and χ^2^ test for categorical variables were used to compare two independent groups. Means were compared using ANOVA test for more than 2 groups. For nonparametric data, Mann-Whitney U-test was used to compare quantitative variables between two groups. Correlations between continuous variables and ordinal parameters were calculated by Pearson’s coefficient and Spearman’s coefficient, respectively. For all of the above, a p-value <0.05 was considered statistically significant and a p-value <0.001 was considered highly statistically significant.

Bivariate logistic regression analysis was used to detect the association between serum OPN levels, considered as a continuous variable, and all possible ordinal variables. The presence of diabetes was categorized as: 0=absence and 1=presence. The presence of DR was categorized as: 0=absence of DR and 1=non-proliferative DR. Diabetic nephropathy (DN) was categorized as 0=absence of DN and 1=presence of persistent microalbuminuria in at least two of three urine samples collected over 24 h.

## RESULTS

Our study included 60 patients with T1DM (Male/Female: 42/18) of a mean age of 11.8±2.2 years, with a mean diabetes duration of 6.1±1.6 years (range 4-10 years) and 60 healthy subjects (Male/Female: 39/21, age 12±2.2 years) as a control group. Clinical and biochemical characteristics of the study population are shown in Table 1. Patients with T1DM had significantly lower BMI, higher FBG, and higher HbA1c values than the control group. The clinical and biochemical findings of patients with T1DM revealed no significant gender difference in diabetes duration, daily insulin dose, BMI, SBP, DBP, HbA1c, and lipid profile.

The overall mean value for HbA1c in patients with T1DM was 8.2±1.5 (8.1±1.2 in males and 8.6±2.2 in females). Acceptable HbA1c (≤8%) values for glycemic control were found in 40% (24 out of 60) of the subjects.

In our subjects, the prevalence of DR in the form of non-proliferative DR was 25% (n=15/60). In this group, diabetes duration was 7±1.5 years and HbA1c (mean ± standard deviation): 8.6±1.4%. Prevalence of DN in the form of microalbuminuria was 21.6% (n=13/60) in patients with a diabetes duration of 7.7±1.9 years and the mean HbA1c value in this group was 9.6±1.6%. We found that 15% (9/60) of T1DM patients had combined DR and DN. There was no significant gender difference regarding DR and DN in patients with T1DM. The prevalence of antihypertensive agents (ACE-I) use was 20% (12/60) in a group with a with diabetes duration of 7.3±1.6 years and with a SBP value of 118±2 mmHg.

Patients with T1DM had significantly higher serum OPN levels compared with controls (13.7±3.4 μg/L vs. 8.9±2.9 μg/L, p<0.001). However, no significant gender difference regarding OPN levels were found in patients with T1DM. Serum OPN concentrations were higher in patients with microalbuminuria than patients with normal albumin excretion rate (AER) and controls. OPN concentrations were also higher in patients with normal AER than the control group (Table 2).

Similarly, patients with retinopathy had higher OPN concentrations than those without (16.8±2 vs. 12.4±3 mg/L; p=0.005). Patients with retinopathy without signs of nephropathy (normal AER) had higher serum OPN concentrations than those who did not have retinal pathology (16.3±2 mg/L vs. 12.4±3 mg/L; p=0.02).

In T1DM patients (n=60), serum OPN levels correlated with higher SBP, DBP, and BMI, lower HDL, and microalbuminuria. However, no correlation was found of OPN levels with HbA1c, insulin dose, and diabetes duration in T1DM patients (Table 3).

To rule out an influence of ACE-I treatment on OPN levels, serum OPN levels were also evaluated separately in T1DM patients according to the use of ACE-Is and no difference was detected between T1DM patients with (n=12) and without ACE-I therapy (n=48). Mean OPN value in T1DM patients treated with ACE-I was 16.4±2.3 mg/L, while mean OPN value in T1DM patients not treated with ACE-I was 15.2±18.5 mg/L (p=0.74).

Finally, the bivariate logistic regression analysis demonstrated that higher serum OPN levels were associated with the diagnosis of T1DM independent of all possible confounders. Also, OPN was independently associated with the development of retinopathy and microalbuminuria in patients with T1DM (Table 4).

## DISCUSSION

T1DM is a heterogeneous disorder characterized by autoimmune-mediated destruction of pancreatic beta cells culminating in absolute insulin deficiency (12).

Our study demonstrated that serum OPN levels are significantly higher in pediatric patients with T1DM compared to healthy participants. This finding is in agreement with the findings of Karamizadeh et al (13) who found increased serum OPN levels in pediatric patients with T1DM compared with healthy children in an Iranian study. Also, our findings are in accordance with previous studies on adult T1DM patients (14). Aspord et al (15) studied the pattern of expression of 524 immune-related genes in the Langerhans cells of diabetic mice and found OPN as one of the early genes that was expressed in the primary phases of diabetes and concluded that OPN may play a vital role in T1DM. Also, OPN has been found as one of the auto-antigens which are expressed by human islet somatostatin cells (16). Moreover, previous studies described OPN as a key regulator of adipose tissue inflammation as both serum levels and adipose tissue expression of some pro-inflammatory cytokines such as tumor necrosis factor alpha, interleukin-6, and inducible nitric oxide synthase were significantly reduced in mice lacking the OPN gene (17). Other studies concluded that OPN deficiency led to reduced adipose tissue inflammation and increased insulin sensitivity (17,18).

Frequency of cardiovascular diseases increases in diabetic patients by two- to fourfold. They are the most common cause of death in patients with T1DM. However, the risk is low before age 30 years (19,20). Risk factors that independently increase cardiovascular risk in patients with diabetes include hypertension, dyslipidemia, renal dysfunction, and hyperglycemia (21).

The current study revealed that serum OPN levels directly correlate with several cardio-metabolic risk factors, such as higher BMI, SBP, DBP, lower HDL, diagnosis of T1DM, but not with insulin dose, diabetes duration, and HbA1c. The only previous study done for assessment of OPN in pediatric patients by Karamizadeh et al (13) did not investigate the presence of clinical and biochemical determinants of high OPN levels. However, our findings are in agreement with a previous study conducted on adult patients with T1DM which demonstrated that circulating OPN levels directly correlate with higher BMI, SBP, and DBP, and lower HDL, but not with diabetes complications, insulin dose, C-peptide level, or disease duration (14). Moreover, the serum OPN levels directly correlated with microalbuminuria. Similar results were reported by Gordin et al (22) who also found an association between higher OPN levels and microalbuminuria at baseline in a cohort study conducted on a large population of adult patients with T1DM. On the other hand, Barchetta et al (14) did not find a significant correlation between serum OPN and microalbuminuria.

In the present work, multivariate logistic regression analyses revealed an association of increased OPN levels with diabetes, DR, and DN, independent from clinical and biochemical confounders. This finding is in agreement with a previous study conducted in adult T1DM patients (14). Also, Gordin et al (22), in a 10-year follow-up study on a cohort of adult patients with T1DM, demonstrated that serum OPN level was an independent predictor of DN, of cardiovascular events, and of all-cause mortality. This finding can be explained by the ability of OPN to increase the pro-inflammatory cytokines in these tissues.

In the present study, only 40% (24 out of 60) of the subjects were found to have achieved acceptable HbA1c levels indicating good glycemic control. This is a serious situation and needs to be underlined since in T1DM patients, poor metabolic control is usually due to inadequacies in insulin regimens and these patients are at highest risk for complications and cardiovascular disorders even at an early age (23).

The main limitation of the present study was the relatively small number of patients participating in the study. Also, the single-center approach limits making generalizations from the results of the study. However, we consider this present study as a baseline for a future cohort study which we plan to conduct on a large population of pediatric patients with T1DM presenting to our endocrinology unit.

In conclusion, this study shows that increased OPN levels are independently associated with T1DM in pediatric patients and identify patients with an unfavorable metabolic profile. Therefore, our data provide a support to the hypothesis that OPN may have a role in the prediction of microvascular diabetes complications. Future studies are needed to demonstrate the clinical benefit of OPN as a possible novel marker of vascular dysfunction and a useful tool for risk stratification in pediatric patients with T1DM.

## Acknowledgments

We express our gratitude to our assistance team and our patients.

## Ethics

Ethics Committee Approval: Institutional Ethical Committee of Zagazig University; September 2014, Informed Consent: Written informed consent was obtained from the parents of the patients involved in the study as recommended by the Institutional Ethics Committee of Zagazig University and in accordance with the Helsinki declaration after a full explanation of the purpose and nature of all procedures used.

Peer-review: Externally peer-reviewed.

## Figures and Tables

**Table 1:**
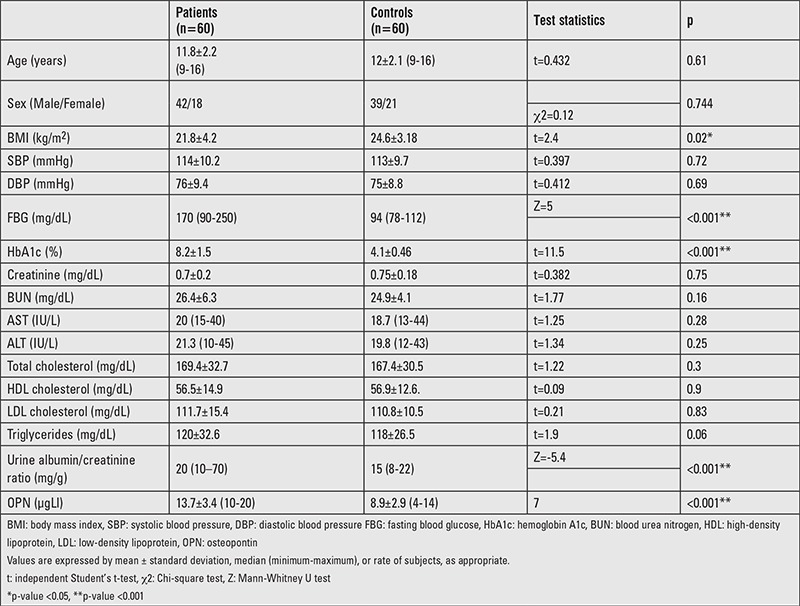
Clinical and biochemical characteristics in type 1 diabetes mellitus patients and in the controls

**Table 2:**
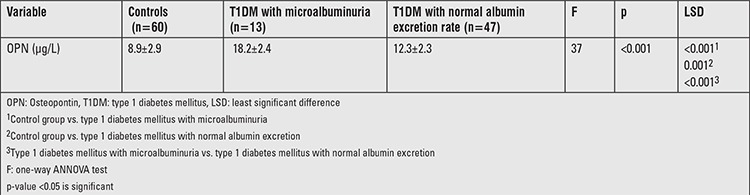
Comparison of osteopontin levels between controls and patients subgroups

**Table 3:**
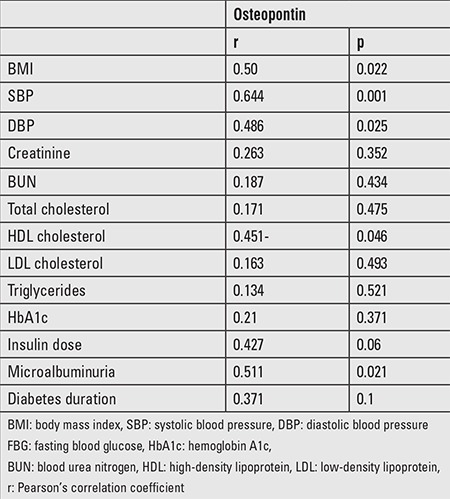
Correlations of osteopontin with clinical and biochemical characteristics of the type 1 diabetes mellitus group

**Table 4:**
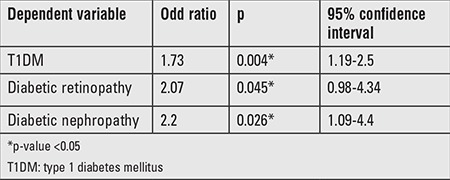
Logistic regression analysis for osteopontin as an independent predictor of type 1 diabetes mellitus, diabetic retinopathy, and nephropathy
